# Robust Polyimide/Graphene Composites as Electrically Functional Materials for Space-Compatible Biomedical Applications

**DOI:** 10.3390/polym18182222

**Published:** 2026-09-12

**Authors:** Martina Campanella, Francesca Blondelli, Elisa Toto, Susanna Laurenzi, Maria Gabriella Santonicola

**Affiliations:** 1 Department of Chemical Engineering Materials Environment, Sapienza University of Rome, Via del Castro Laurenziano 7, 00161 Rome, Italy; martina.campanella@uniroma1.it (M.C.); francesca.blondelli@uniroma1.it (F.B.); elisa.toto@uniroma1.it (E.T.); 2 Sapienza Aerospace Structures Laboratory, Sapienza University of Rome, Via Salaria 851, 00138 Rome, Italy; susanna.laurenzi@uniroma1.it

**Keywords:** polyimide composites, graphene, dry electrodes, UV-C sterilization, surface analysis

## Abstract

Polyimide-based composites with graphene nanoplatelet (GNP) fillers are investigated as multifunctional materials for potential space-compatible biomedical monitoring. These composites are designed to combine the intrinsic high thermal and chemical stability of polyimides, as well as their well-established radiation resistance reported in the literature, with the electrical functionality of graphene. PI/GNP membranes are fabricated by casting dispersions of graphene nanoplatelets within the polyimide solution, using a green and bio-based solvent (dimethyl isosorbide), followed by thermal treatment in vacuum. The effect of UV-C irradiation is investigated as a sterilization-relevant stress condition, showing that the PI/GNP composites retain their main chemical and surface characteristics after exposure. In addition, irradiation is associated with an improvement in the electrical response, suggesting that the conductive network formed by graphene nanoplatelets remains effective after treatment. Overall, this study highlights the PI/GNP composites as robust and electrically functional candidate materials for dry electrode applications in harsh environments. By combining chemical stability, thermal resistance, a stable surface and electrical conductivity, these membranes emerge as promising candidates for future health monitoring applications on Earth and during long-term missions in space.

## 1. Introduction

Human space exploration is increasingly oriented toward long-duration missions, where astronauts are exposed to extreme environmental conditions, including microgravity, cosmic radiation, vacuum, and wide temperature variations [1,2]. These factors pose significant risks to astronaut health, potentially leading to physiological alterations in the cardiovascular, neurological, and musculoskeletal systems [3,4]. In this context, continuous health monitoring is fundamental to preserving astronaut safety and well-being, and to facilitating early diagnosis during space missions [3,5]. Reliable monitoring systems are relevant for future human exploration scenarios, where autonomous health assessment and reduced dependence on Earth-based medical support will be essential [6].

Wearable sensors must meet demanding requirements related to reliability, radiation and thermal resistance, mechanical flexibility, low weight, and user comfort, while also complying with strict mass and volume constraints [3,7]. These aspects become particularly critical during extended missions, where sensor materials should operate for prolonged periods with limited maintenance and minimal generation of consumable waste. Conventional Ag/AgCl electrodes are poorly suited for prolonged missions due to gel dehydration, skin irritation, limited stability and waste generation [8,9]. Consequently, the development of dry electrodes represents a key requirement for wearable sensors in space applications. At the same time, dry electrodes are also relevant for terrestrial wearable biomedical monitoring, where gel-free, stable, and comfortable electrode materials are required for long-term, repeated, or remote signal acquisition. For this purpose, candidate materials must not only provide stable electrical performance and skin compatibility but also tolerate sterilization and decontamination procedures.

UV-C exposure is widely adopted for surface sterilization in biomedical devices and represents a non-contact, low-mass-compatible approach potentially suitable for future human spaceflight [10]. However, UV-C radiation may induce surface oxidation, degradation phenomena, or variations in electrical properties, depending on the chemical structure and composition of the material [11,12]. Therefore, evaluating the response of candidate electrode materials after UV-C irradiation is essential to assess their suitability for biomedical monitoring systems intended for both terrestrial use and space-related operating conditions. These requirements point toward the use of lightweight multifunctional materials that are able to combine electrical functionality, environmental stability and durability.

Polymer-based materials and composites have attracted increasing interest in aerospace and space applications because of their low density, processability, tunable properties, and potential multifunctionality. In particular, polymer systems have been investigated for radiation shielding, lightweight structural and functional components, and in-space manufacturing strategies [13,14,15,16,17]. The incorporation of conductive fillers into high-performance polymer matrices further enables the design of electrically conductive materials suitable for sensing and monitoring applications under demanding operating conditions [18,19,20].

Among high-performance polymers, aromatic PIs represent particularly promising candidates for space-compatible devices [21,22]. They are known for high mechanical and chemical stability, radiation resistance, and remarkable optical performance [23]. The high glass transition temperature and wide thermal stability range make them suitable for operation under extreme conditions such as those encountered in space environments [24,25]. Consequently, these polymers have been extensively employed in space applications and represent suitable matrices for the development of multifunctional composite membranes. Previous studies have highlighted the potential of polyimide membranes for lightweight space devices [26,27], as well as the importance of more sustainable routes for the synthesis of high-performance PI materials [28]. Moreover, their bio-inertia and biocompatibility have increasingly enabled their use in biomedical applications [29].

Graphene-based polymer composites have been widely investigated for wearable electronic devices and sensing applications, including biomedical, environmental, and human health monitoring [30,31]. When incorporated into polymer matrices, graphene can promote the formation of conductive pathways while preserving the flexibility and processability of the host material. Graphene/polymer composites have therefore been widely investigated for sensing applications, including environmental and human health monitoring [32,33,34].

In this study, polyimide/graphene membranes with different GNP filler concentrations and tailored electrical conductivity are developed. The aim is to combine the robustness of polyimides, which are widely used in space applications because of their well-established stability in space environments, with the electrical functionality required for candidate dry electrode materials for wearable biomedical monitoring, including both terrestrial and spaceflight scenarios. Polyimide synthesis and membrane fabrication were carried out using dimethyl isosorbide (DMI), a green, bio-based non-toxic solvent [35]. This processing approach is consistent with the growing interest in reducing the environmental impact associated with the fabrication of high-performance polymers for advanced technologies. Fourier transform infrared (FTIR) spectroscopy was employed to investigate the chemical structure of the composite membranes and to evaluate the degree of imidization. The surface morphology was investigated by scanning electron microscopy (SEM), and quantitative roughness parameters were extracted from SEM images using MountainsMap software. Surface wettability was evaluated through static water contact angle measurements. Considering the temperature variations encountered in space environments, like in low Earth orbit (LEO), the thermal behavior of the membrane was analyzed. Electrical performance was investigated through frequency-dependent impedance measurements. The properties were also evaluated after UV-C exposure to assess the stability of the membranes after a sterilization-relevant treatment. The UV-C irradiation was selected exclusively as a sterilization-relevant condition and was not intended to reproduce the ionizing radiation environment encountered in space.

Overall, the results provide insight into the potential of the PI/GNP membranes as candidate materials for dry electrodes for wearable biomedical monitoring on Earth and during future human space missions.

The novelty of the present work lies in the combination of a green processing approach based on DMI with a comprehensive investigation of the chemical, thermal, morphological and electrical stability of PI/GNP membranes after UV-C irradiation as a sterilization-relevant condition for potential biomedical applications in space environments.

## 2. Materials and Methods

### 2.1. Materials

Diamine 4-BDAF (Tokyo Chemical Industry, Tokyo, Japan; purity > 98%) and dianhydride 6FDA (Alfa Aesar, Kandel, Germany; purity 97%) were used as monomers. Dimethyl isosorbide (DMI, purity 97.5%), a green and non-toxic solvent with a boiling point of 240 °C at 1 atm, was purchased from Sigma-Aldrich (Milan, Italy). Acetic anhydride (purity > 99%) and pyridine (purity > 99.9%) were supplied by Chem-Lab (Zedelgem, Belgium) and Honeywell Riedel-de Haën (Charlotte, NC, USA), respectively. Graphene nanoplates grade AO-4 (Graphene Supermarket, Calverton, NY, USA) with specific surface area ≤ 40 m^2^/g, purity 98.5%, average flake thickness 60 nm, and particle lateral size ≤ 7 µm were employed as a nanofiller. Ultrapure water (resistivity 18.2 MΩ-cm) was obtained from a Direct-Q3 UV water purification system (Millipore, Molsheim, France). All chemicals were used as received without further purification.

### 2.2. Polyimide Synthesis and Solution Preparation

The polyimide was synthesized using a process optimized in previous works [28,36]. Briefly, the poly(amic acid) (PAA) was obtained by reacting the aromatic and fluorinated dianhydride (6FDA) with the diamine (4-BDAF) under argon atmosphere at room temperature. Specifically, a stoichiometric amount of 6FDA was added to a mechanically stirred solution of 4-BDAF in dimethyl isosorbide (DMI) and then stirred for 24 h at room temperature. This solution was prepared with a solid content of 15% by weight (wt%). The chemical imidization was carried out using pyridine and acetic anhydride and further stirring of the solution for 24 h at room temperature under argon atmosphere. The mixture was then precipitated in ultrapure water under stirring, then filtered and milled. The polyimide powder was subsequently purified with ultrapure water and dried in an oven for 4 h at 115 °C to remove residual water, followed by 4 h at 200 °C under vacuum for evaporation of DMI solvent residues. The dried polyimide powder was dissolved in DMI at a concentration of 15 wt% and stirred for 48 h to obtain the polyimide solution (PI).

### 2.3. Fabrication of Polyimide/Graphene Membranes

Different amounts of GNPs were added to the polyimide solution and dispersed using an ultrasonic bath (Elmasonic P30H, Elma, Singen, Germany) for 2 h. The dispersions obtained were mechanically stirred at 45 °C for 8 h. The PI/GNP dispersions were prepared at the following concentrations of GNP: 5 wt%, 10 wt%, 15 wt% and 20 wt%. The 10, 15 and 20 wt% GNP formulations were selected as the main composition investigated in this study, while the 5 wt% formulation was additionally considered for selected characterization to further investigate the influence of GNP loading at a lower concentration.

**Figure 1:**
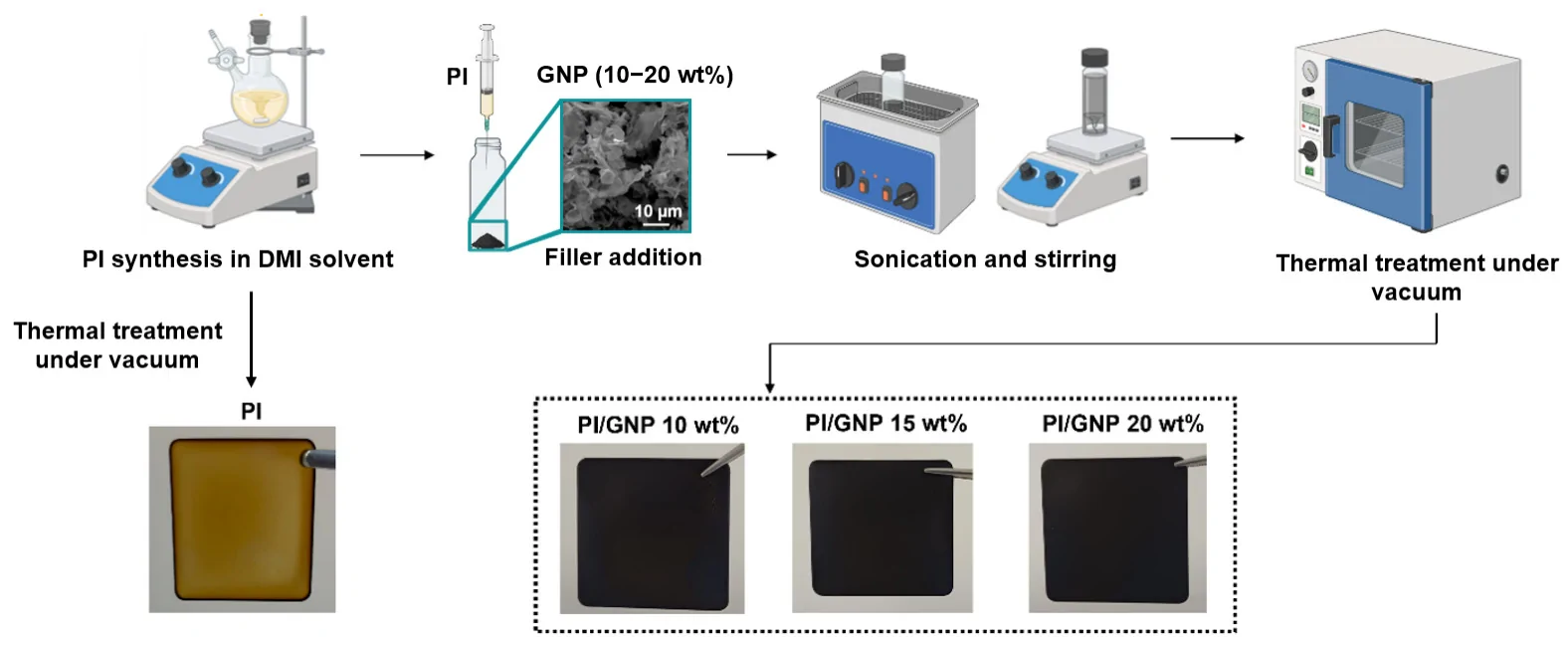
Schematic representation of the preparation of PI solution and PI/GNP dispersions (10–20 wt% of GNP). PI and PI/GNP membranes are fabricated by solution casting followed by thermal treatment up to 200 °C under vacuum.

The PI solution and PI/GNP dispersions were cast into a silicon mold (size: 3 cm × 3 cm) and heat-treated in a vacuum oven (WOV-30, Witeg, Wertheim, Germany). The thermal treatment, applied to remove the solvent, involved a gradual temperature increase from 25 °C to 100 °C at a rate of 1 °C/min, followed by a 1 h hold at 100 °C under vacuum. Next, the temperature was raised from 100 °C to 200 °C at the same rate, with a subsequent 3 h hold at 200 °C under vacuum. This thermal profile was developed according to an optimized protocol established in our previous work [28]. A schematic representation of the PI/GNP dispersion preparation and membrane fabrication process is shown in Figure 1. The thickness of the obtained membranes ranged from 99 μm for pure PI to 126 μm for the PI/GNP composite containing 20 wt% graphene.

### 2.4. Sterilization by UV-C

The effect of UV-C irradiation on pristine PI and PI/GNP composites was investigated as a sterilization-relevant stress condition. The PI-based membranes were irradiated in a UV-Sterilizer (YM-9003, DM Soluzioni ICT, Pescara, Italy) under a UV lamp (power 5 W, voltage 220–240 V, frequency 50 Hz) at a wavelength of 254 nm, at room temperature and 35% relative humidity. Samples were positioned 15 cm from the lamp and exposed to a UV-C irradiance of 17.7 W/m^2^. The irradiation test was performed for 1 h, corresponding to a total radiation dose of 63.7 kJ/m^2^. The applied dose is comparable to or higher than those reported in the literature for effective UV-C sterilization [37,38,39].

### 2.5. Characterization Methods

The chemical structures of the PI and PI/GNP composites were investigated using a Nicolet Summit FTIR Spectrometer (Thermo Fisher Scientific, Waltham, MA, USA) in attenuated total reflectance (ATR) mode with a diamond crystal. For each spectrum, 64 scans at a spectral resolution of 4 cm^−1^ in the wavenumber range 4000–500 cm^−1^ were co-added and analyzed using the OMNIC Software (version 2.0, Thermo Fisher Scientific, Waltham, MA, USA) provided with the instrument. FTIR spectra were used to calculate the degree of imidization (ID) of the PI and PI/GNP samples, following the method proposed by Chen et al. [40] using Equation (1):

(1) $$ID=\frac{\frac{A_{1376}}{A_{1501}}}{\frac{A_{1376(300{}^{\circ}C)}}{A_{1501(300{}^{\circ}C)}}}\times 100$$

In particular, the ID was determined from the ratio between the absorption band at 1376 cm^−1^, associated with the C–N stretching of the imide group, and the band at 1501 cm^−1^, assigned to the aromatic C=C stretching and used as an internal reference. The obtained ratio was normalized with respect to that of the fully imidized membranes, which was obtained by applying an additional thermal treatment at 300 °C for 1 h after the standard membrane preparation protocol. The fully imidized reference membranes were used only for the normalization procedure and are not included in the results. For each formulation, ID measurements were performed on at least three independent spectra of the same formulation. The ID values reported correspond to the average of these independent measurements and the standard deviation was below 1%. The mean absorbance values obtained from the three ATR-FTIR spectra before and after UV-C exposure, together with those of the corresponding 300 °C reference samples, are reported in Table S1.

Sample morphologies were investigated by scanning electron microscopy (SEM), using a VEGA II LSH instrument (Tescan, Brno, Czech Republic), with an accelerating voltage of 10 kV. SEM images of the sample surfaces were acquired at a magnification of 500× and used for the stereoscopic reconstruction and the surface roughness analysis. The acquired SEM images had a field of view of approximately 577 μm and a lateral pixel size of 0.56 μm/pixel. Additional SEM images focusing on the cross-section of the samples were acquired at magnification of 2500×. Before the SEM analysis, samples were coated with gold using a Cressington Sputter Coater 108auto (Cressington Scientific Instruments, Watford, UK), which deposits a conductive layer under an argon inert atmosphere. All samples were coated for 15 s. Surface roughness measurements were obtained from SEM images using MountainsMap software (version 10.2.3, Digital Surf, Besançon, France). The software calculates roughness parameters in accordance with ISO 21920 [41], comparing two SEM images acquired at 0 degree and at tilt angles of 1 degree. A Gaussian S-filter with a cut-off value of 2.5 μm and a Gaussian L-filter with a cut-off value of 0.25 mm were applied during the surface roughness analysis. The stereoscopic reconstruction was not intended to resolve nanoscale surface features, but rather to provide a comparative assessment of surface topography at microscale. In this work, the arithmetic mean roughness (R_a_) and the root mean square roughness (R_q_) were considered [42]. For each sample, four non-overlapping regions of interest (ROIs) 200 × 200 μm, extracted from a minimum of three different SEM images, were analyzed. For each ROI, approximately three hundred 2D profiles were extracted by the MountainsMap software, and from these profiles the surface roughness parameters were calculated according to ISO 21920. The final reported values correspond to the average of the roughness parameters obtained from four ROIs per sample type. For the comparison of surface roughness before and after UV-C exposure, Welch’s two-sample *t*-test was performed separately for R_a_ and R_q_ for each formulation, using a significance level of α = 0.05. The results of the statistical analysis are reported in Table S2 of the Supporting Information.

Thermal analysis was performed by differential scanning calorimetry using a DSC 8500 (PerkinElmer, Waltham, MA, USA) to determine the glass transition temperature (T_g_). Before DSC analysis, all samples were pre-treated in a laboratory oven at 300 °C for 30 min and then allowed to cool to room temperature. The following DSC thermal cycle was applied: the sample was heated from 25 °C to 300 °C (heating rate: 10 °C/min), held at 300 °C for 5 min, cooled down to 25 °C (cooling rate: 10 °C/min), held at 25 °C for 5 min and then reheated to 300 °C (heating rate: 10 °C/min). DSC samples (ca. 10 mg) were sealed in aluminum pans and measurements were performed under a constant nitrogen flow (40 mL/min). T_g_ values were determined from the second heating DSC thermograms at the inflection point in the curves using the Thermal Analysis Software (version 13.3.1.0014, PerkinElmer, Waltham, MA, USA) provided with the instrument, in accordance with the ASTM International standards E1356-08 and D3418-15 [43,44].

Surface wettability was evaluated by measuring the water contact angle (WCA) on the membrane surface using an optical analyzer (OCA15Pro, DataPhysics Instruments, Filderstadt, Germany). WCA values were determined by drop shape analysis using the DataPhysics SCA 20 software module (version 4.5.8). For each sample formulation, a minimum of three different samples were analyzed. Ten droplets of degassed ultrapure water (3 μL each) were deposited on different areas of each sample and the results averaged.

The electrical properties of the PI/GNP membranes with different graphene concentrations were investigated over the frequency range 20–1000 Hz using a precision Agilent E4980A LCR meter (Agilent Technologies, Santa Clara, CA, USA). A Teflon cell containing two rectangular copper electrodes in contact with the sample surface was employed for the measurements. For each formulation, the electrical measurements were carried out on a minimum of three different samples. The LCR meter determined the resistance and capacitance of the samples. Once these values were obtained, the impedance of the samples was calculated by assuming an equivalent parallel RC circuit, according to the following equation:

(2) $$\left|Z_{RC}\right|=\frac{1}{\sqrt{{\left(\frac{1}{R}\right)}^{2}+{\left(2\pi fC\right)}^{2}}}$$

The impedance values obtained in this work refer to the intrinsic electrical response of the PI/GNP membranes and do not represent the electrode–skin interfacial impedance that occurs during signal acquisition. The electrical conductivity (σ) was calculated from the measured resistance as an effective quantity to enable the comparison among the different membrane formulations. Details of the calculation procedure are provided in the Supporting Information.

## 3. Results and Discussion

### 3.1. Analysis of PI and PI/GNP Membranes

**Figure 2:**
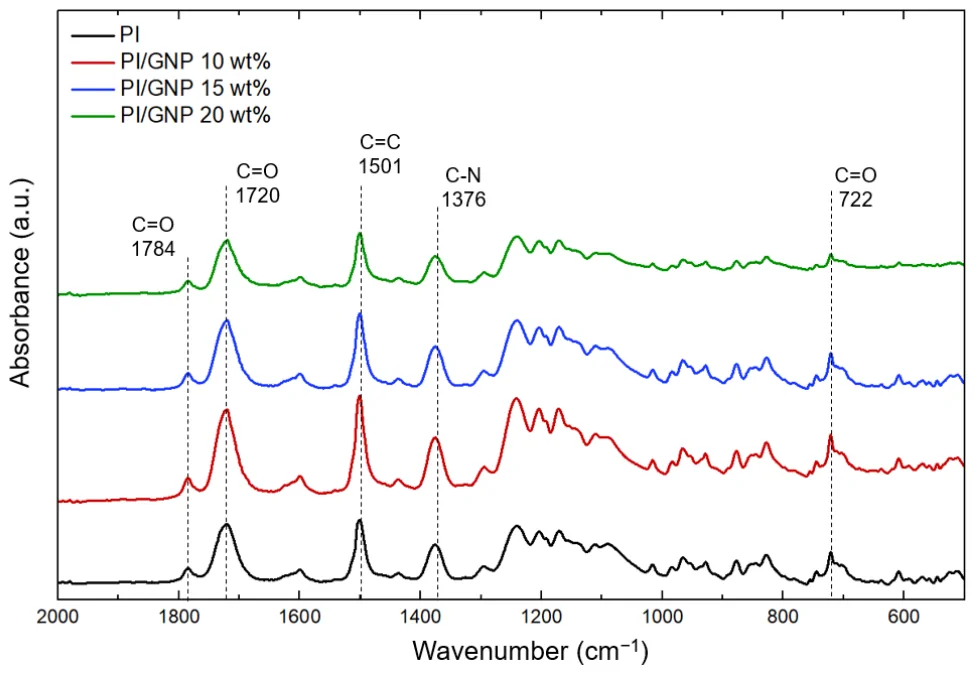
ATR-FTIR spectra of PI and PI/GNP membranes in the fingerprint region (2000–500 cm^−1^). Data are offset for clarity.

The chemical structure of PI and PI/GNP membranes was investigated by FTIR spectroscopy. The corresponding spectra are reported in Figure 2. The spectra exhibit the typical absorption bands associated with imide groups, including the peaks at 1784 and 1720 cm^−1^, assigned to the asymmetric and symmetric stretching vibrations of the carbonyl groups, respectively. The band at 1376 cm^−1^ is attributed to the C–N stretching, while the signal at 722 cm^−1^ corresponds to the C=O bending vibration of the imide rings [45]. In addition, the absorption band observed at 1501 cm^−1^ can be assigned to the C=C stretching vibration of para-substituted benzene rings. The corresponding full-range ATR-FTIR spectra are provided in Figure S2 of the Supporting Information. No absorption bands associated with residual poly(amic acid) were detected at 1660 and 1540 cm^−1^ or above 2500 cm^−1^, either before or after UV-C exposure.

FTIR analysis was also employed to calculate the degree of imidization (ID) of PI and PI/GNP membranes, following the method proposed by Chen et al. [40]. As reported in Table 1, all samples exhibit an ID close to 90%, indicating that the chemical imidization and the applied heat treatment efficiently promote the formation of imide groups and that the incorporation of graphene does not interfere with this process. The ID values obtained in this work are in good agreement with the imidization behavior reported in the literature for aromatic polyimides. Similar degrees of imidization (~90%) have been observed after thermal treatment at approximately 200–250 °C [46].

**Figure 3:**
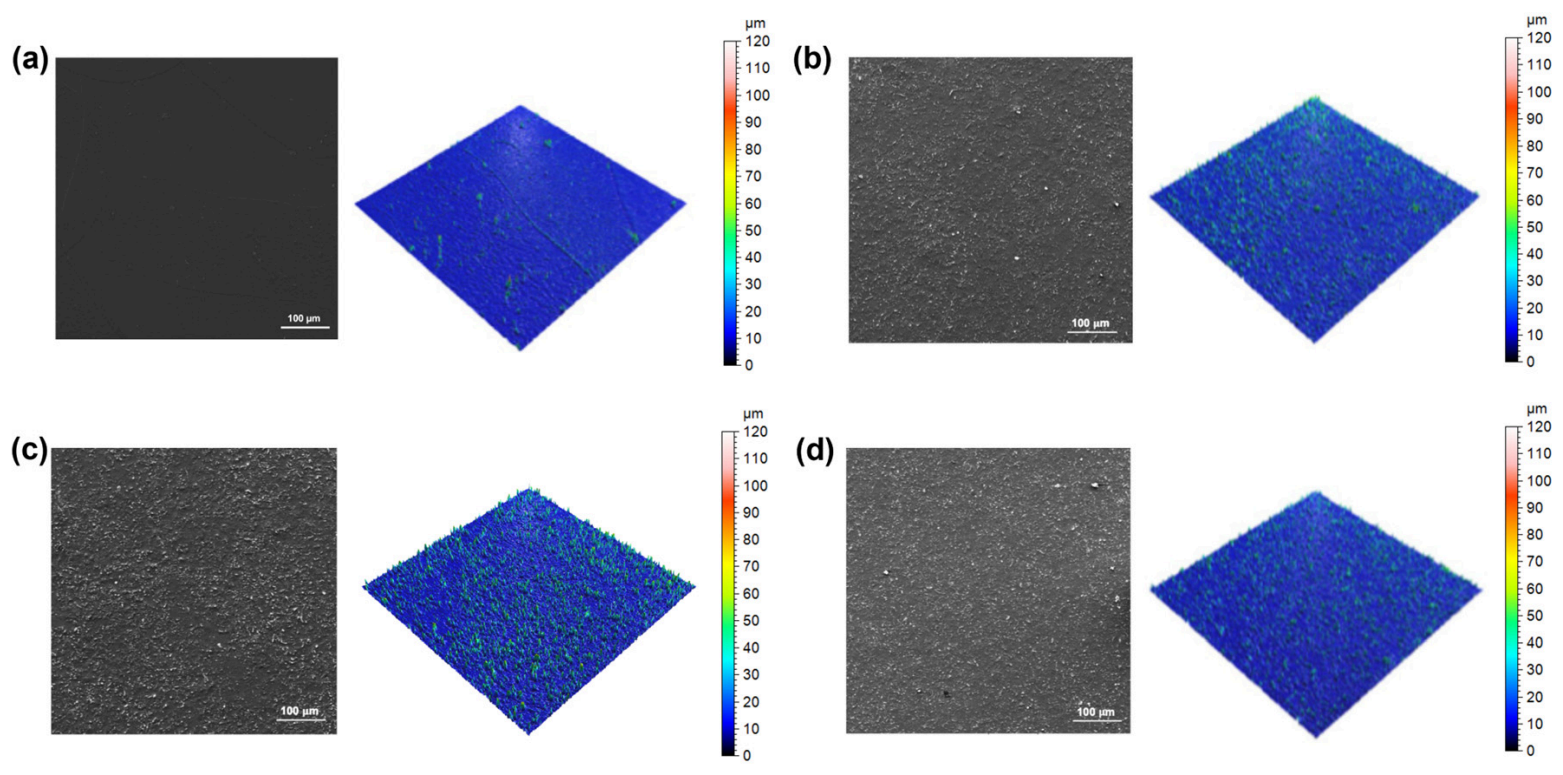
SEM images acquired at magnification of 500× and corresponding 3D-surface reconstruction generated using MountainsMap software of (**a**) neat PI, (**b**) PI/GNP 10 wt%, (**c**) PI/GNP 15 wt%, (**d**) PI/GNP 20 wt%.

**Figure 4:**
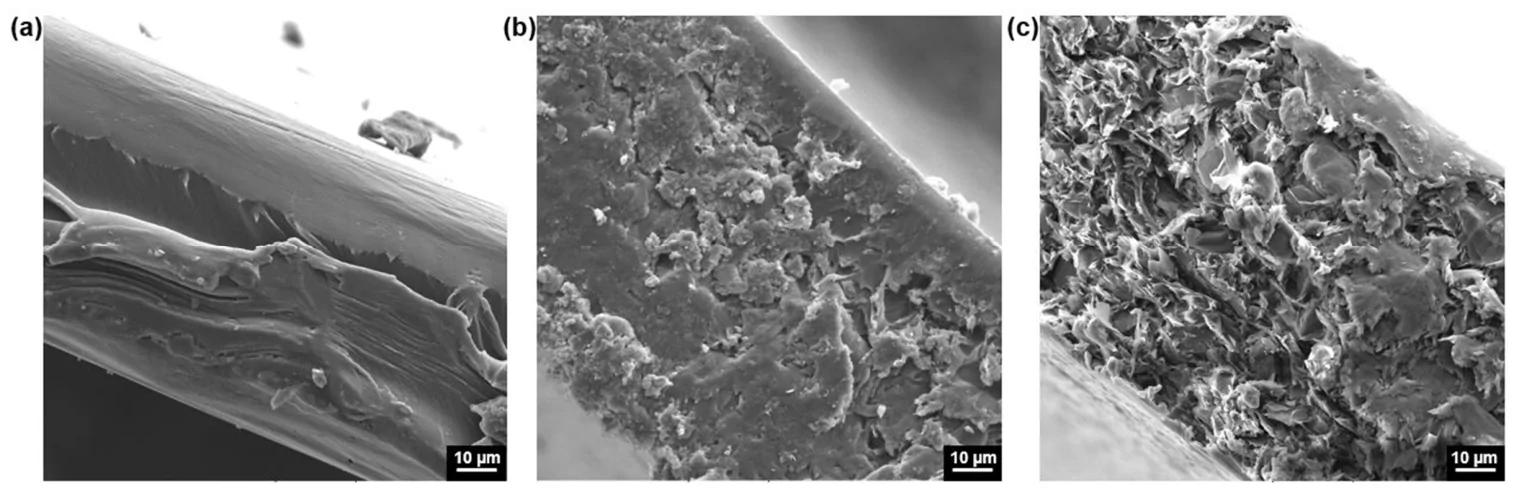
Representative cross-sectional SEM images of (**a**) PI, (**b**) PI/GNP 10 wt% and (**c**) PI/GNP 20 wt% acquired at magnification of 2.5k×.

The morphological and surface roughness analyses for the neat PI and the PI/GNP composite membranes were carried out using SEM images processed by the MountainsMap software. Figure 3 reports SEM images acquired at magnification of 500× and the corresponding three-dimensional surface reconstruction generated by the software. In addition, cross-sectional SEM images of selected membranes are shown in Figure 4 to provide further investigation of the internal morphologies of the composites.

The 3D-surface reconstructions were used to quantitatively evaluate the surface roughness parameters R_a_ and R_q_, as reported in Table 2. The incorporation of GNPs increased the surface roughness compared to pristine polyimide, indicating that graphene modifies the surface morphology of the membranes. Both R_a_ and R_q_ increased from 10 to 15 wt% GNP concentration; no further increase was observed at 20 wt%. These results suggest that the increase in surface roughness tends to level off at higher GNP contents.

The thermal behavior of the membranes was investigated by DSC analysis over the temperature range from 25 °C to 300 °C, specifically to determine the glass transition temperatures (T_g_), which are summarized in Table 3. The corresponding DSC thermograms are provided in Figure S1 of the Supporting Information. A slight increase in T_g_ was observed after the incorporation of GNP, with no substantial differences among the composite membranes. This behavior may be associated with the partial restriction of polyimide chain mobility induced by the presence of graphene nanoplatelets and has also been reported for similar graphene/polyimide composites in the literature [47]. Considering that temperature variations during space missions, most notably in LEO and lunar environments, may reach up to 150 °C [48,49], the T_g_ values obtained for all composite membranes are compatible with the thermal conditions considered for space-relevant operation.

**Figure 5:**
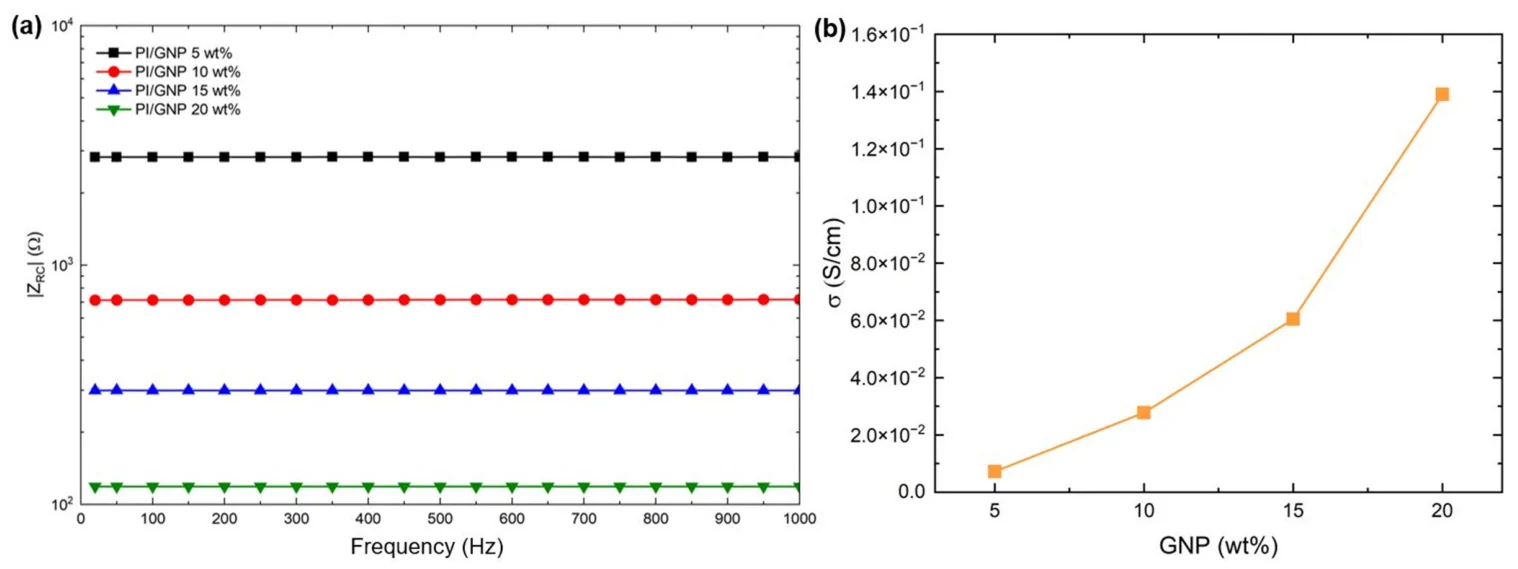
(**a**) Impedance modulus (|Z_RC_|) of PI/GNP composites at different filler loadings as a function of frequency (20–1000 Hz); (**b**) electrical conductivity (σ) of PI/GNP membranes at different GNP concentrations.

The electrical properties of PI/GNP membranes were evaluated through frequency-dependent electrical measurements. The resistance and capacitance of the samples were used to calculate the electrical impedance according to Equation (2). As shown in Figure 5a, the impedance remains constant over the investigated frequency range for all samples, indicating that the capacitive contribution is negligible and that the membranes exhibit resistive behavior. Under this condition, the resistance term in Equation (2) dominates over the capacitive term so the measured impedance is essentially determined by the sample resistance. To further investigate the influence of graphene loading on the electrical response, additional impedance measurements were performed on a PI/GNP membrane containing 5 wt% GNP. This formulation was included exclusively for electrical characterization to provide additional information on the effect of filler concentration at lower graphene contents. The impedance decreases with increasing GNP content, confirming the role of graphene nanoplatelets in improving the electrical conductivity of the polymer matrix [50,51]. The 5 wt% membrane exhibited the highest impedance among the conductive composites, while a progressive decrease in impedance was observed with increasing GNP loading. Composites containing 10 wt% GNP or higher exhibit impedance values consistently below 1 kΩ across the investigated frequency range. These values are in good agreement with the intrinsic impedance and resistance reported in the literature for graphene-based and polymer-based materials employed in bioelectrode systems [52,53].

Since membrane thickness increases with GNP loading, the electrical conductivity (σ)was also calculated as an effective quantity to enable comparison of the electrical properties among the different formulations. As reported in Table 4 and shown in Figure 5b, conductivity increases progressively with graphene content, in agreement with the impedance results. A similar trend has been reported for graphene oxide/polyimide composites, where the conductivity increased with filler loading because of conductive network formation [54].

### 3.2. UV-C Effects on Surface Properties of PI and PI/GNP Composites

**Figure 6:**
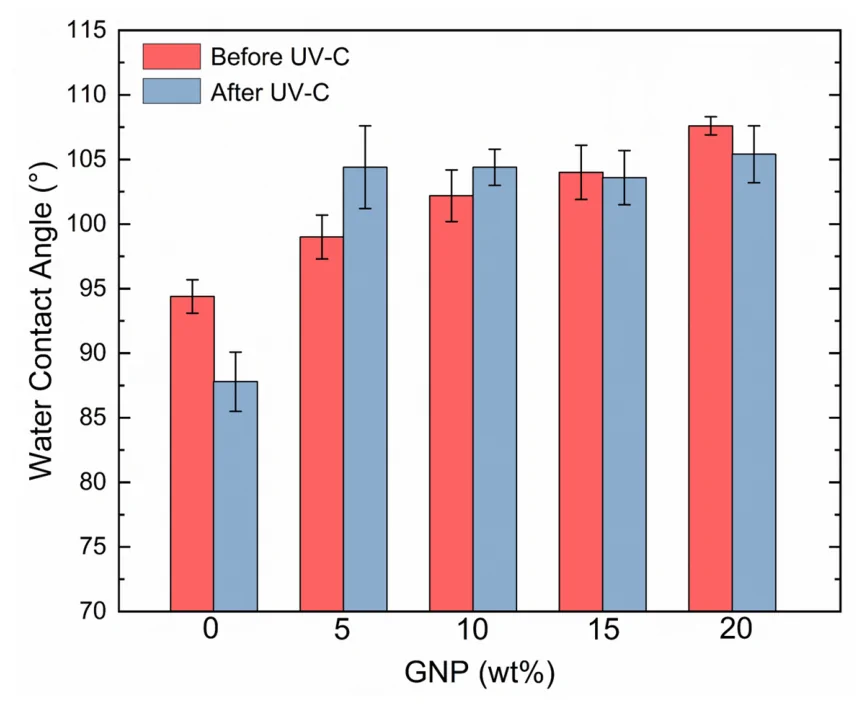
Water contact angle values of PI and PI/GNP membranes before and after UV-C irradiation (UV-C dose of 63.7 kJ/m^2^). Error bars indicate the standard deviation of measurements obtained from three samples per membrane type, with ten replicates for each sample.

The effect of UV-C irradiation on the surface wettability of the PI and PI/GNP membranes was evaluated by water contact angle measurements. Both pristine PI and PI/GNP samples maintain a hydrophobic surface, with WCA values close to or above 90° (Figure 6). Surface wettability was evaluated because it influences the surface characteristics of the membrane and may contribute to the long-term stability of dry electrodes [55]. However, electrode performance also depends on several additional factors, including the electrode–skin interface, which was not investigated in this study.

After 1 h exposure to UV-C, PI membranes show a slight decrease in water contact angles, whereas the PI/GNP membranes exhibit negligible variations. The corresponding WCA values are reported in Table 5, confirming that graphene incorporation helps preserve the surface properties of the membranes under UV-C irradiation.

**Figure 7:**
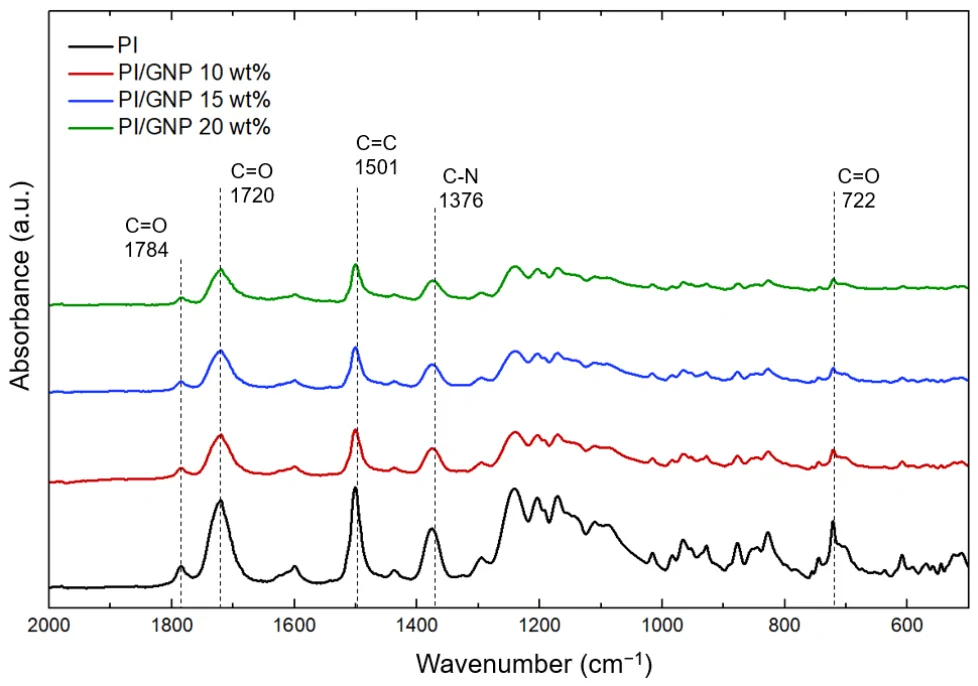
ATR-FTIR spectra of UV-C exposed PI and PI/GNP membranes in the fingerprint region (2000–500 cm^−1^). Data are offset for clarity. Samples were exposed to a UV-C dose of 63.7 kJ/m^2^.

The FTIR spectra of the UV-C-exposed samples are reported in Figure 7. The corresponding full-range ATR-FTIR spectra after UV-C exposure are reported in Figure S3 of the Supporting Information. Compared with the corresponding non-irradiated samples (Figure 2), both pristine PI and PI/GNP membranes show no qualitative spectral changes after UV-C irradiation. In particular, no new absorption bands and relevant peak shifts are observed, suggesting that UV-C exposure does not induce major chemical modifications, such as oxidation or degradation. This behavior can be mainly attributed to the intrinsic UV resistance of polyimide, while the presence of graphene nanoplatelets may further contribute to limiting photo-induced degradation phenomena. Nevertheless, a variation in the relative intensities of 1376 and 1501 cm^−1^ bands is detected, resulting in the increase in ID.

The degree of imidization after UV-C irradiation was calculated from the FTIR spectra according to the method proposed by Chen et al. [40]. As shown in Table 6, ID values close to 98% were obtained for all membranes. The increase in the degree of imidization after UV-C irradiation may be attributed to photo-induced cyclization of residual amic acid groups, which promotes the formation of additional imide rings and leads to a higher overall imidization level [56].

**Figure 8:**
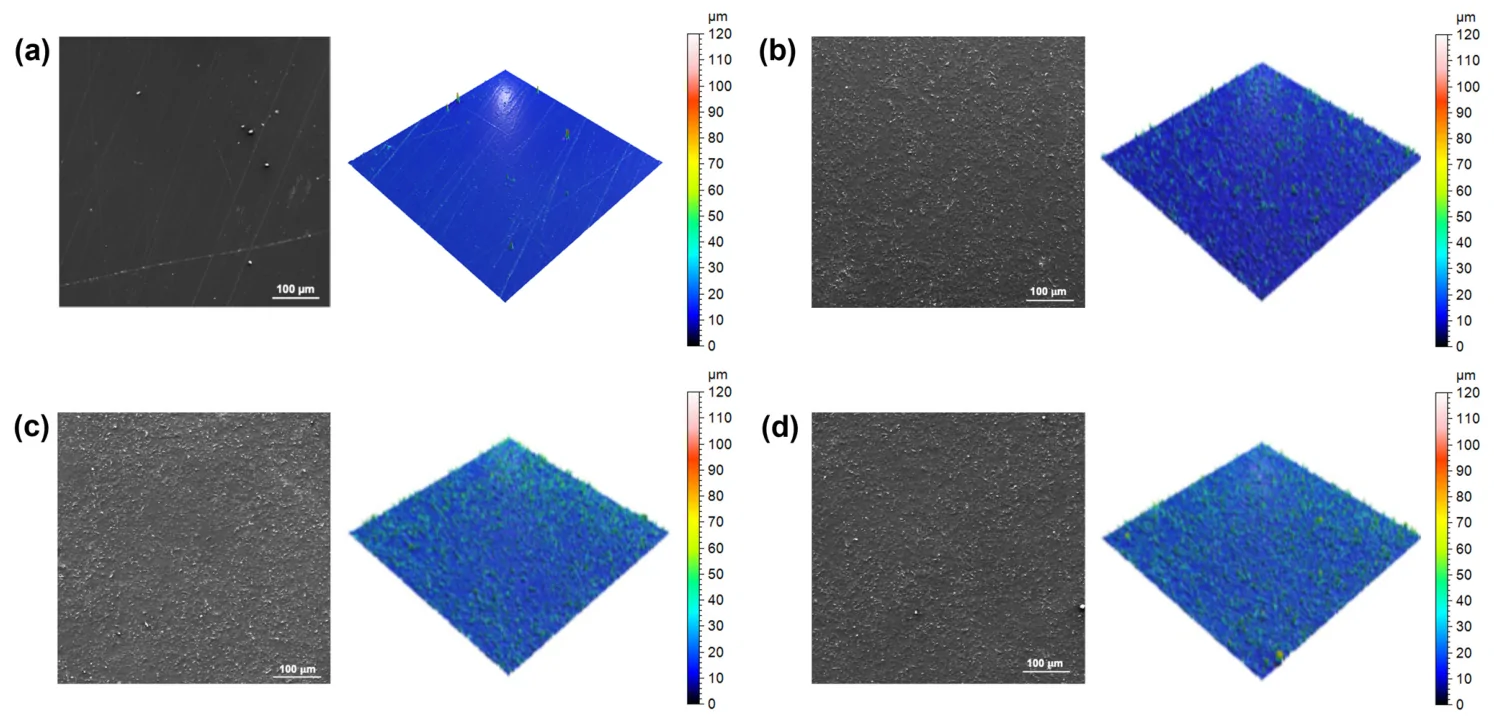
SEM images and corresponding 3D surface reconstruction by MountainsMap software of (**a**) PI, (**b**) PI/GNP 10 wt%, (**c**) PI/GNP 15 wt%, (**d**) PI/GNP 20 wt% samples after UV-C irradiation (UV-C dose of 63.7 kJ/m^2^).

Figure 8 shows the SEM images of the UV-C exposed PI and PI/GNP membranes and the corresponding 3D surface reconstructions carried out by the MountainMap software. The roughness parameters are summarized in Table 7.

To investigate whether the changes in the roughness parameters before and after UV-C exposure were statistically significant, a Welch’s two-sample *t*-test was performed. Overall, the measured R_a_ and R_q_ values remained comparable to those of the corresponding non-irradiated membranes. The full results can be found in the Supporting Information (Table S2): no statistically significant differences in the roughness parameters were determined after UV-C irradiation for neat PI, PI/GNP 10 wt%, PI/GNP 20 wt% for either Ra or Rq (*p* > 0.05). For the PI/GNP 15 wt% membranes, a statistically significant decrease was observed for R_a_ whereas the difference in R_q_ was not statistically significant. Despite the statistical significance observed for R_a_, the absolute variation was relatively small, and the roughness values remained within the same overall range. These results suggest that the membranes retain their surface morphological characteristics upon UV-C exposure, indicating a good morphological stability of both pristine PI and PI/GNP membranes under the applied irradiation conditions.

**Figure 9:**
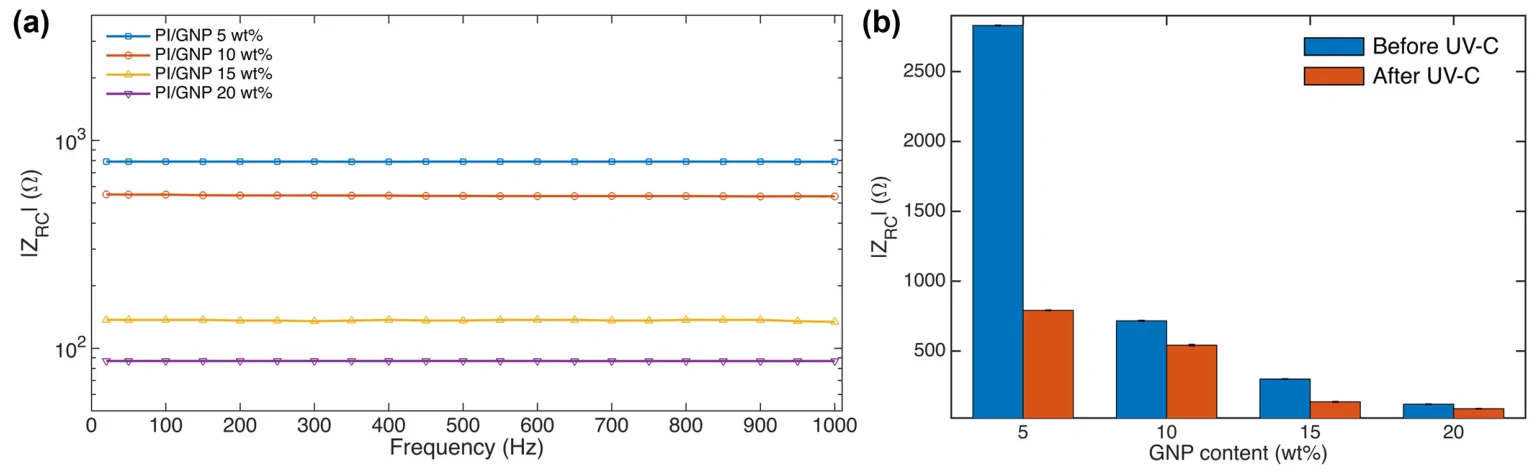
(**a**) Impedance modulus (|Z_RC_|) of PI/GNP composite samples with different filler loadings as a function of frequency (20–1000 Hz) after UV-C irradiation. (**b**) Comparison of the impedance modulus of PI and PI/GNP samples before and after UV-C exposure at a dose of 63.7 kJ/m^2^.

The electrical properties of the membranes were evaluated after UV-C exposure by analyzing their impedance response. Figure 9a shows the impedance magnitude as a function of frequency, while the corresponding values are summarized in Figure 9b. Here, error bars represent the standard deviation of the measurements and are very small, indicating high reproducibility of the experimental data. A decrease in the impedance is generally observed after UV-C treatment, with the most pronounced variation (~257%) occurring for the PI/GNP membranes with 5 wt% of GNP. For the membranes with higher GNP content, the change in electrical properties does not follow a monotonic trend, going from 36% for the PI/GNP 10 wt% to 121% for the PI/GNP 15 wt% and 37% for the PI/GNP 20 wt% samples. Overall, UV-C exposure does not impair the electrical response of the membranes, as the impedance remains low and stable over the investigated frequency range.

The slight reduction in impedance and the corresponding increase in electrical conductivity (Table 8) after UV-C exposure can be ascribed to irradiation-induced modifications occurring in the surface region of the polyimide matrix. In particular, the ATR-FTIR analysis indicated an increase in the ID after UV-C exposure which may be associated with the observed increase in conductivity at the membrane surface. This interpretation is limited to the surface region (a few microns in depth) and does not imply a modification of the bulk of the membranes. In addition, UV irradiation has been reported to induce graphitic structural transformation in polyimide under appropriate irradiation conditions [57]. While no direct evidence of such transformations was obtained in the present work, they may have contributed to the increase in electrical conductivity after UV-C exposure. Overall, the electrical performance of the composite membranes was not impaired by UV-C exposure, while their morphological stability was preserved.

## 4. Conclusions

Polyimide/graphene (PI/GNP) composite membranes were successfully fabricated and characterized as potential dry electrode materials for biomedical signal monitoring in both terrestrial and space environments. Aromatic and fluorinated polyimide membranes containing 10–20 wt% graphene nanoplatelets were obtained using DMI as a greener solvent alternative. The membranes exhibited high degrees of imidization, hydrophobic surfaces (water contact angle close to or above 90°), and glass transition temperatures ranging from 253 °C for the neat polyimide samples to about 262 °C for the composite ones. These results support their potential suitability for space-relevant operating conditions, including in LEO and lunar environments. Surface morphology analysis showed that the incorporation of graphene increased roughness compared to pristine polyimide, while the roughness values remained comparable among the PI/GNP membranes. Electrical characterization demonstrated that increasing the GNP content progressively reduced the impedance and increased the electrical conductivity, while the membranes exhibit resistive behavior over the investigated range. UV-C treatment was used as a sterilization-relevant stress condition to evaluate the response of the membranes. After UV-C exposure, no significant changes were observed in chemical composition, wettability, or surface roughness. A decrease in impedance was detected after irradiation, indicating that UV-C exposure did not impair the electrical response of the materials. The impedance remained frequency-independent, indicating predominantly resistive behavior after UV-C as well. Overall, the PI/GNP composites combine chemical stability, high thermal resistance, stable surface properties, and suitable electrical performance. These properties highlight the potential use of the PI/GNP membranes as support materials for dry electrodes for wearable biomedical monitoring in spaceflight and terrestrial applications. Further studies evaluating the electrode–skin interface, mechanical flexibility and bending fatigue, as well as long-term aging under space-relevant radiation exposure will be required to fully assess their suitability for long-duration space applications.

## Figures and Tables

**Table 1:** Degree of imidization (ID) of PI and PI/GNP composites calculated from the ratio between the absorption bands at 1376 and 1501 cm^−1^. Values represent the mean of at least three independent FTIR measurements (standard deviation < 1%).

Samples	ID
PI	91.8%
PI/GNP 10 wt%	92.6%
PI/GNP 15 wt%	92%
PI/GNP 20 wt%	89.8%

**Table 2:** Surface roughness parameters calculated by MountainsMap software. Values are reported as mean ± standard deviation (SD) of four non-overlapping 200 × 200 µm ROIs extracted from a minimum of three different SEM images per sample.

Sample	R_a_ (µm)	R_q_ (µm)
PI	1.06 ± 0.12	1.65 ± 0.24
PI/GNP 10 wt%	2.28 ± 0.79	3.64 ± 1.28
PI/GNP 15 wt%	4.04 ± 0.28	6.14 ± 0.34
PI/GNP 20 wt%	3.84 ± 0.17	5.82 ± 0.27

**Table 3:** Glass transition temperatures of PI and PI/GNP membranes determined by DSC after sample pre-treatment at 300 °C for 30 min. Values represent the mean ± the standard deviation obtained from three different samples for each formulation.

Samples	T_g_ (°C)
PI	253.54 ± 0.26
PI/GNP 10 wt%	260.51 ± 1.04
PI/GNP 15 wt%	262.59 ± 1.12
PI/GNP 20 wt%	262.24 ± 2.25

**Table 4:** Electrical conductivity of PI/GNP composite membranes with different GNP concentrations before UV-C irradiation.

Samples	σ (S/cm)
PI	-
PI/GNP 5 wt%	0.723 × 10^−2^ ± 0.001 × 10^−2^
PI/GNP 10 wt%	2.812 × 10^−2^ ± 0.078 × 10^−2^
PI/GNP 15 wt%	6.023 × 10^−2^ ± 0.035 × 10^−2^
PI/GNP 20 wt%	13.890 × 10^−2^ ± 0.116 × 10^−2^

**Table 5:** Water contact angles for PI and PI/GNP membranes at different concentrations of GNP before and after UV-C exposure (UV-C dose of 63.7 kJ/m^2^). Data were averaged over a minimum of three different samples of the same type, with ten replicates per sample.

Sample	WCA (°)	
	Before UV-C	After UV-C
PI	94.4 ± 1.3	87.8 ± 2.3
PI/GNP 5 wt%	99.0 ± 1.7	104.4 ± 3.2
PI/GNP 10 wt%	102.2 ± 2.0	104.4 ± 1.4
PI/GNP 15 wt%	104.0 ± 2.1	103.6 ± 2.1
PI/GNP 20 wt%	107.6 ± 0.7	105.4 ± 2.2

**Table 6:** Degree of imidization (ID) of UV-C exposed PI and PI/GNP membranes, calculated from the ratio between the absorption bands at 1376 and 1501 cm^−1^. Values represent the mean of at least three independent FTIR measurements (standard deviation < 1%).

Samples	ID
PI	98.7%
PI/GNP 10 wt%	98.3%
PI/GNP 15 wt%	98.2%
PI/GNP 20 wt%	97.6%

**Table 7:** Surface roughness parameters calculated by MountainsMap software after UV-C exposure. Values are reported as mean ± standard deviation (SD) of four non-overlapping 200 × 200 µm ROIs extracted from a minimum of three different SEM images per sample.

Samples	R_a_ (µm)	R_q_ (µm)
PI	1.01 ± 0.07	1.90 ± 0.15
PI/GNP 10 wt%	2.79 ± 0.51	4.43 ± 0.80
PI/GNP 15 wt%	3.45 ± 0.33	5.36 ± 0.54
PI/GNP 20 wt%	3.72 ± 0.45	5.86 ± 0.73

**Table 8:** Electrical conductivity of PI/GNP composite membranes with different GNP concentrations after UV-C irradiation.

Samples	σ (S/cm)
PI	-
PI/GNP 5 wt%	2.583 × 10^−2^ ± 0.006 × 10^−2^
PI/GNP 10 wt%	3.660 × 10^−2^ ± 0.047× 10^−2^
PI/GNP 15 wt%	13.280 × 10^−2^ ± 0.114 × 10^−2^
PI/GNP 20 wt%	19.040 × 10^−2^ ± 0.040 × 10^−2^

## Data Availability

The original contributions presented in this study are included in the article/Supplementary Material. Further inquiries can be directed to the corresponding author.

## Supplementary Materials

The following supporting information can be downloaded at https://www.mdpi.com/article/10.3390/polym18182222/s1, S1: Calculation of electrical conductivity; Figure S1: Zoomed-in view of the DSC thermograms used to determine the glass transition temperature of neat PI and PI/GNP membranes following thermal pre-treatment at 300 °C for 30 min. Heat flow is normalized by the sample weight; Figure S2: ATR-FTIR spectra of neat PI and PI/GNP membranes with different GNP concentrations. Data are offset for clarity; Figure S3: ATR-FTIR spectra of neat PI and PI/GNP membranes with different GNP concentrations after exposure to UV-C radiation with a dose of 63.7 kJ/m^2^. Data are offset for clarity; Table S1: Absorbance values at 1376 and 1501 cm^−1^ obtained from averaging three ATR-FTIR spectra for each membrane before and after UV-C exposure, together with the corresponding absorbance values of the reference samples thermally treated at 300 °C. The degree of imidization was calculated individually for each spectrum using the corresponding A_1376_ and A_1501_ values and subsequently averaged over the three spectra. Standard deviations of the absorbance values are negligible; Table S2: Statistical comparison of SEM-derived surface roughness parameters (R_a_ and R_q_) before and after UV-C exposure for neat PI and PI/GNP composite membranes. Values are reported as mean and standard deviation (SD) and were calculated from the analysis of four non-overlapping regions of interest (ROIs) per sample type. Statistical comparisons between pre- and post-UV-C measurements were performed using Welch’s two-sample *t*-test, with a significance level of α = 0.05. Statistically significant differences were observed only for R_a_ of the PI/GNP 15 wt% membrane, while no significant differences were determined for the other samples.
