# Supplementary material for: Robust Polyimide/Graphene Composites as Electrically Functional Materials for Space-Compatible Biomedical Applications
Source: Polymers (Basel). 2026 Sep 12;18(18):2222. doi: 10.3390/polym18182222 (PMC13611752; doi:10.3390/polym18182222)
Supplement: Supplementary file 1 [file polymers-18-02222-s001.zip › polymers-4429911-supplementary.pdf]

Supporting Information

Robust Polyimide/Graphene Composites as Electrically Functional Materials for Space-Compatible Biomedical Applications

Martina Campanella [1], Francesca Blondelli [1], Elisa Toto [1], Susanna Laurenzi [2] and Maria Gabriella Santonicola [1,*]

[1] Department of Chemical Engineering Materials Environment, Sapienza University of Rome, Via del Castro Laurenziano 7, 00161 Rome, Italy; martina.campanella@uniroma1.it (M.C.); francesca.blondelli@uniroma1.it (F.B.); elisa.toto@uniroma1.it (E.T.)
[2] Sapienza Aerospace Structures Laboratory, Sapienza University of Rome, Via Salaria 851, 00138 Rome, Italy; susanna.laurenzi@uniroma1.it
* Correspondence: mariagabriella.santonicola@uniroma1.it

Experimental details

S1. Calculation of electrical conductivity

The sheet conductivity (σ_s) of the membranes was calculated from the measured resistance R using the following Equation:

$$\sigma_S = (R \times \frac{D}{L})^{-1}$$

where D is the length of the electrode in contact with the sample and L is the distance between the two electrodes. With the experimental setup used in this study, D is 2.2 cm and L is 0.5 cm. The electrical conductivity (σ) was subsequently obtained by dividing the sheet conductivity by the membrane thickness (t):

$$\sigma = \frac{\sigma_S}{t}$$

The membrane thicknesses were 99, 102, 105, 115, and 126 μm for neat PI and PI/GNP membranes containing 5, 10, 15, and 20 wt% GNP, respectively. The resulting electrical conductivity values were expressed in S/cm and used for comparison among the different membrane formulations.

Supplementary Figures and Tables

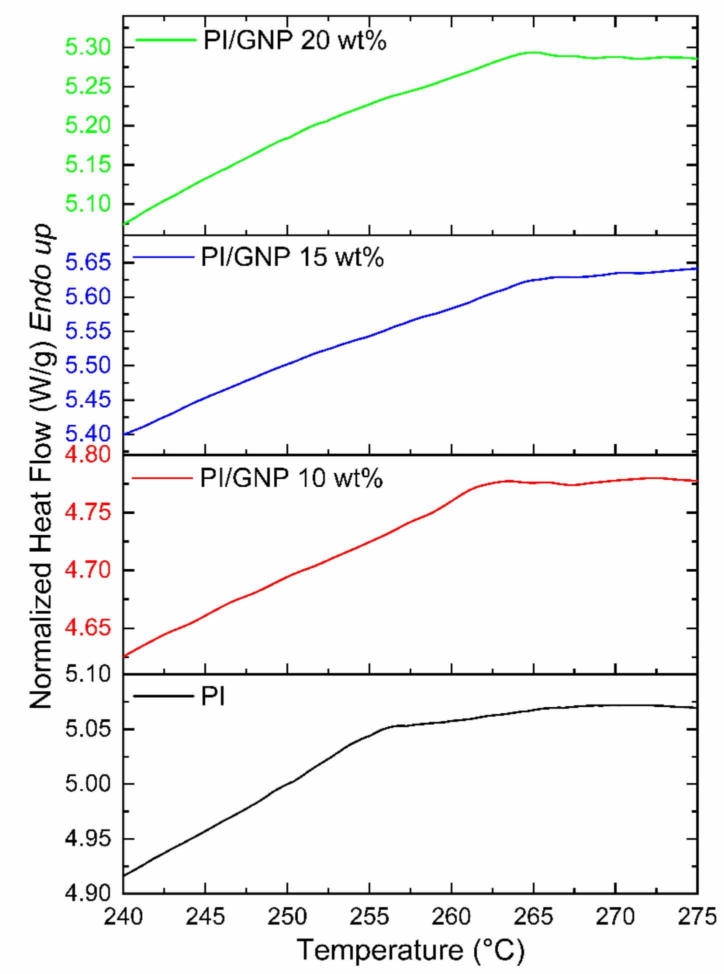

Figure S1. Zoomed-in view of the DSC thermograms used to determine the glass transition temperature of neat PI and PI/GNP membranes following thermal pre-treatment at 300 °C for 30 min. Heat flow is normalized by the sample weight.

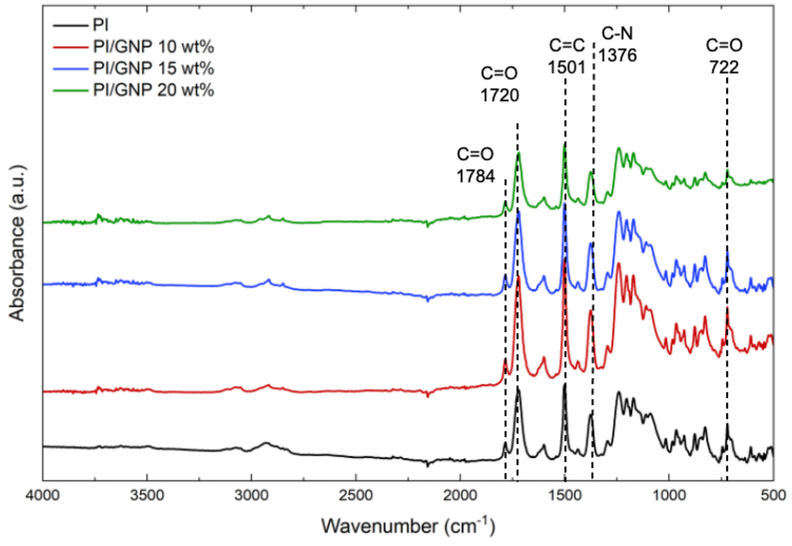

Figure S2. ATR-FTIR spectra of neat PI and PI/GNP membranes with different GNP concentrations. Data are offset for clarity.

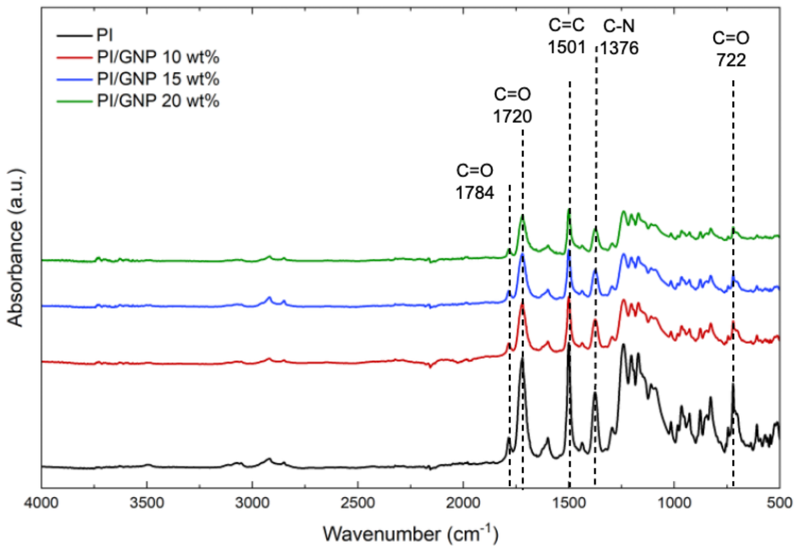

Figure S3. ATR-FTIR spectra of neat PI and PI/GNP membranes with different GNP concentrations after exposure to UV-C radiation with a dose of 63.7 kJ/m². Data are offset for clarity.

Table S1. Absorbance values at 1376 and 1501 cm^{-1} obtained from averaging three ATR-FTIR spectra for each membrane before and after UV-C exposure, together with the corresponding absorbance values of the reference samples thermally treated at 300 °C. The degree of imidization was calculated individually for each spectrum using the corresponding A_{1376} and A_{1501} values and subsequently averaged over the three spectra. Standard deviations of the absorbance values are negligible.

	**Pre UV-C**		**Post UV-C**		**Reference at 300 °C**	
	A_{1376}	A_{1501}	A_{1376}	A_{1501}	A_{1376}	A_{1501}
**PI**	0.0344	0.0654	0.0350	0.0619	0.0181	0.0316
**PI/GNP 10 wt%**	0.0212	0.0447	0.0081	0.0160	0.0185	0.0360
**PI/GNP 15 wt%**	0.0292	0.0638	0.0594	0.1217	0.0111	0.0223
**PI/GNP 20 wt%**	0.0524	0.1224	0.0164	0.0354	0.0082	0.0172

Table S2. Statistical comparison of SEM-derived surface roughness parameters (R_a and R_q) before and after UV-C exposure for neat PI and PI/GNP composite membranes. Values are reported as mean and standard deviation (SD) and were calculated from the analysis of four non-overlapping regions of interest (ROIs) per sample type. Statistical comparisons between pre- and post-UV-C measurements were performed using Welch's two-sample t-test, with a significance level of $\alpha = 0.05$. Statistically significant differences were observed only for R_a of the PI/GNP 15 wt% membrane, while no significant differences were determined for the other samples.

**Sample**	**Parameter**	**Mean pre UV-C (μm)**	**SD pre UV-C (μm)**	**Mean post UV-C (μm)**	**SD post UV-C (μm)**	**p-value**	**Significant**
**PI**	R_a	1.06	0.12	1.01	0.07	0.508	No
**PI**	R_q	1.65	0.24	1.90	0.15	0.131	No
**PI/GNP 10wt%**	R_a	2.28	0.79	2.79	0.51	0.327	No

**PI/GNP 10 wt%**	R_q	3.64	1.28	4.43	0.80	0.344	No
**PI/GNP 15 wt%**	R_a	4.04	0.28	3.45	0.33	0.036	Yes
**PI/GNP 15 wt%**	R_q	6.14	0.34	5.36	0.54	0.058	No
**PI/GNP 20 wt%**	R_a	3.84	0.17	3.72	0.45	0.619	No
**PI/GNP 20 wt%**	R_q	5.82	0.27	5.86	0.73	0.917	No
